# The impact of elevated C-reactive protein level on the prognosis for oro-hypopharynx cancer patients treated with radiotherapy

**DOI:** 10.1038/s41598-017-18233-w

**Published:** 2017-12-19

**Authors:** Atsuto Katano, Wataru Takahashi, Hideomi Yamashita, Kentaro Yamamoto, Mizuo Ando, Masafumi Yoshida, Yuki Saito, Osamu Abe, Keiichi Nakagawa

**Affiliations:** 1 Department of Radiology, The University of Tokyo, 7-3-1 Hongo, Bunkyo-ku, Tokyo 113-8655 Japan; 2 Department of Radiology, Japan Self Defense Force Central Hospital, 1-2-24 Ikejiri, Setagaya-ku, Tokyo 154-8532 Japan; 3 Department of Otolaryngology-Head and Neck Surgery, The University of Tokyo, 7-3-1 Hongo, Bunkyo-ku, Tokyo 113-8655 Japan

## Abstract

The purpose of this study was to investigate an association between the prognosis for oro-hypopharynx squamous cell carcinoma treated with radiation therapy and the pre-therapeutic level of C-reactive protein (CRP). Patient with oro-hypopharyngeal squamous cell carcinoma who underwent definitive radiotherapy in our institution from January 2002 to August 2016 were enrolled. The patient were divided into elevated CRP (over 0.3 mg/dl) group and normal CRP groups, according to pre-treatment serum levels. There were 276 evaluable patients, and the median follow up was 41 months, ranging from 2 to 171 months. The 3-year OS and CSS for all enrolled patients were 67.0% and 72.8%, respectively. The OS and CSS rates were significantly worse in the elevated CRP group than in the normal CRP group, according to Kaplan-Meier survival curves analysed by a Log-rank test (p = 0.005 and p < 0.001, respectively). Multivariate analyses indicated that serum CRP levels remained independent predictors for both OS (HR: 1.588, p = 0.022) and CSS (HR: 1.989, p = 0.005). The pre-treatment CRP level is an independent predictor of treatment prognosis in patients with oro-hypopharyngeal cancer who underwent definitive radiotherapy. Especially, it is curious that an elevated CRP serum level is a significant predictor of loco-regional recurrence.

## Introduction

C-reactive protein (CRP) belongs to a family of acute-phase proteins whose plasma concentrations increase in response to inflammation. When inflammation or destruction of tissue cells occurs in the body, CRP is secreted from the liver^1^ into the blood. From the Virchow’s hypothesis in 1863 it has been revealed that there is a close relationship between inflammation and cancer^2–4^. It is known that chronic inflammation causes carcinogenesis and progression of cancer^5^. Parkin *et al*. suggested that about 20% of malignant tumours are closely related to chronic inflammation associated with infection^6^. In particular, the relationship between Helicobacter pylori, Epstein-Barr virus, human papilloma virus, hepatitis virus and cancer is well known. Many studies have shown the elevation of pre-treatment CRP to be a significant prognostic indicator in patients with esophageal cancer^7^, non-small cell lung cancer^8^ hepatocellular carcinoma^9^, renal cell cancer^10^ and prostate cancer^11^. Although various factors have been suggested as prognostic indicators in cancer patient, measurements of CRP seem to be a fast, simple and cost-effective predictor in clinical practice.

Radiation therapy is a feasible and effective modality in the treatment of head and neck squamous cell cancer, especially in regard to organ preservation. Platinum-based chemo-radiotherapy, which is regarded as standard treatment in locally advanced cases, may be administered in two ways: induction or concurrent chemotherapy. As induction chemotherapy, a combination of cisplatin and 5-fluorouracil (CF regimen) has long been regarded as a standard in locally advanced head and neck cancer, however, in the TAX323 trial evaluating docetaxel, platinum plus 5-fluorouracil (DCF regimen) was superior to the CF regimen in survival and function preservation^12^. Concurrent chemotherapy is also beneficial in all tumor subsites in head and neck cancer. The results of the Radiation Therapy Oncology Group 91–11 study revealed that cisplatin-radiotherapy is superior in loco regional control and larynx preservation compared with induction chemotherapy^13^. Nevertheless, severe late toxicity and high treatment-related deaths after concurrent chemotherapy are important issues to overcome^14,15^. Conclusive recommendations for chemo-radiotherapy in head and neck cancer have not been well established. It is important to quickly elucidate the factors that determine the prognosis because it will allow choosing the most appropriate treatment modality.

The prognostic role of CRP in patients with head and neck cancer is not fully understood. The purpose of this study was to investigate the relationship between serum CRP level and prognosis in oro-hypopharynx cancer patients treated by radiotherapy.

## Materials and Methods

We included oro-hypopharyngeal squamous cell carcinoma patients treated with definitive radiotherapy in our hospital from January 2002 to August 2016. Written informed consent was obtained from all subjects. This study was performed in accordance with the guidelines approved by the institutional review board at the University of Tokyo Hospital. Clinical staging was performed according to the 7th edition of American Joint Committee on Cancer (AJCC) staging in oropharyngeal and hypopharyngeal cancer. All patients enrolled in this study satisfied the following eligibility criteria: (a) histologically confirmed primary squamous cell carcinoma; (b) no evidence of distant metastasis; (c) radiotherapy for definitive intent, excluding palliative intent cases; and (d) no history of previous radiotherapy to oropharynx or hypopharynx.

All patients underwent radiotherapy with 6–10 MV photon linear accelerators. The prescription dose to primary lesions or positive nodes ranged from 57 to 72 Gy (median 66 Gy). Prophylactic nodal areas were irradiated at doses of 40–56 Gy. The dose-fractionation regimen is either once daily, twice daily or concomitant boost. The radiation method was either three-dimensional conformal radiotherapy or intensity modulated radiotherapy (IMRT), including volumetric modulated arc therapy (VMAT). Radiotherapy was combined with induction and/or concurrent chemotherapy, mostly consisting of a platinum-based regimen, although targeted therapy such as cetuximab was also used. We excluded post-operative radiotherapy cases, such as total laryngectomy or pharyngo-laryngectomy with thyroidectomy resulting in positive resection margins, or extra-capsular extension of lymph nodes or partial resections such as trans-oral surgery. Unilateral or bilateral neck dissections alone were included in our study, according to previous report criteria^16^. Pre-therapeutic CRP levels and hemoglobin concentration were measured in peripheral venous blood samples. Anemia was defined as hemoglobin concentration < 11 g/dl, known as a predictor of tumor hypoxia^17^. The normal serum level of CRP was defined as 0.3 mg/dL or lower, according to several references^18,19^. Comparisons between two groups were calculated with chi-square or fisher exact test for qualitative data. For prognosis analysis, we examined the factors of the primary site, age, gender, KPS, smoking history, current smoking status, clinical stages, anemia, pre-therapeutic CRP level and radiation treatment modalities.

All statistical analyses were performed with the R statistical package (The R Foundation for Statistical Computing, Vienna, Austria). Overall survival (OS) and cause-specific survival (CSS) were measured from the first day of initial therapy. Loco-regional control (LRC) was measured from the first day of initial therapy until the date of local or regional relapse, even if distant metastasis occurred with no evidence of local-regional failure. Cases lost to follow-up or death from any cause were regarded as censored cases. Distant metastasis control (DMC) was defined as the period until detection of first distant metastasis without considering loco-regional relapse. The OS, CSS, LRC and DMC rates were calculated by the Kaplan-Meier method using the Log-Rank test. Univariate and multivariate analyses were considered significant at p < 0.05. We conducted a multivariate Cox proportional hazard analysis by using covariates with p < 0.10 in the univariate analysis.

## Results

Among 303 consecutive eligible patients, we excluded 27 cases (8.9%) without any predictor, smoking data were missing for 3 patients, and CRP data were missing for 24 patients. We conducted statistical analyses on the remaining 276 patients. All patients had no evidence of active infection, inflammatory disease, trauma and heart attack at the time of CRP evaluation.

### Patient characteristics

There were 141 oropharynx patients and 135 hypopharynx patients. TNM staging of each cancer is presented in supplementary table 1. The median age of patients was 65 years, ranging from 21 years to 93 years. The patients were predominantly male (92%) with a 10 pack-year smoking history (75%). Induction chemotherapy consisted mainly of the DCF (docetaxel, platinum plus 5-fluorouracil) regimen followed by the CF (platinum plus 5-fluorouracil) regimen and other regimens, such as CDDP alone, the DC (platinum plus docetaxel) regimen and oral S-1 administration. Concurrent chemotherapy, consisting of the DCF regimen, CF regimen, platinum alone or other regimens, including intra-arterial CDDP, cetuximab, and weekly docetaxel/platinum-based regimens. Other patient characteristics and treatment modalities are summarized in Table 1. The median value of normal CRP was 0.29 mg/dL (interquartile range: 0.08–0.90 mg/dL). A total of 132 patients (48%) were grouped into an elevated CRP group, and 144 patients (52%) were grouped into a normal CRP group.

**Table 1:** Patient characteristics and treatment modality of 276 patient with oropharyngeal and hypopharyngeal squamous cell carcinoma.

Variables	Number [Total 276]	Percentage
**Patient characteristics**		
Site of primary lesion		
Hypopharynx	135	49%
Oropharynx	141	51%
Age		
>65 years old	128	46%
≤65 years old	148	54%
Gender		
Male	253	92%
Female	23	8%
Karnofsky Performance Scale		
≥90	205	74%
<90	71	26%
Current smoker		
Yes	119	43%
No	157	57%
Smoking history		
≥10 packs year	208	75%
<10 packs year	68	25%
Clinical T stage		
4	43	16%
3	79	29%
2	113	41%
1	41	15%
Clinical N stage		
3	23	8%
2	126	46%
1	41	15%
0	86	31%
**Treatment modality**		
Radiation method		
IMRT	53	19%
Conventional 3D-CRT	223	81%
Radiation fractionation		
Once daily	244	88%
Twice daily	8	3%
Concomitant boost	24	9%
Neck dissection		
Yes	41	15%
No	235	85%
Concurrent chemotherapy		
Yes	112	41%
DCF	36	13%
CF	43	16%
Platinum alone	20	7%
Other regimens	13	5%
No	164	59%
Induction chemotherapy		
Yes	93	34%
DCF	80	29%
CF	11	4%
Other regimens	3	1%
No	182	66%

### Survival

**Figure 1:**
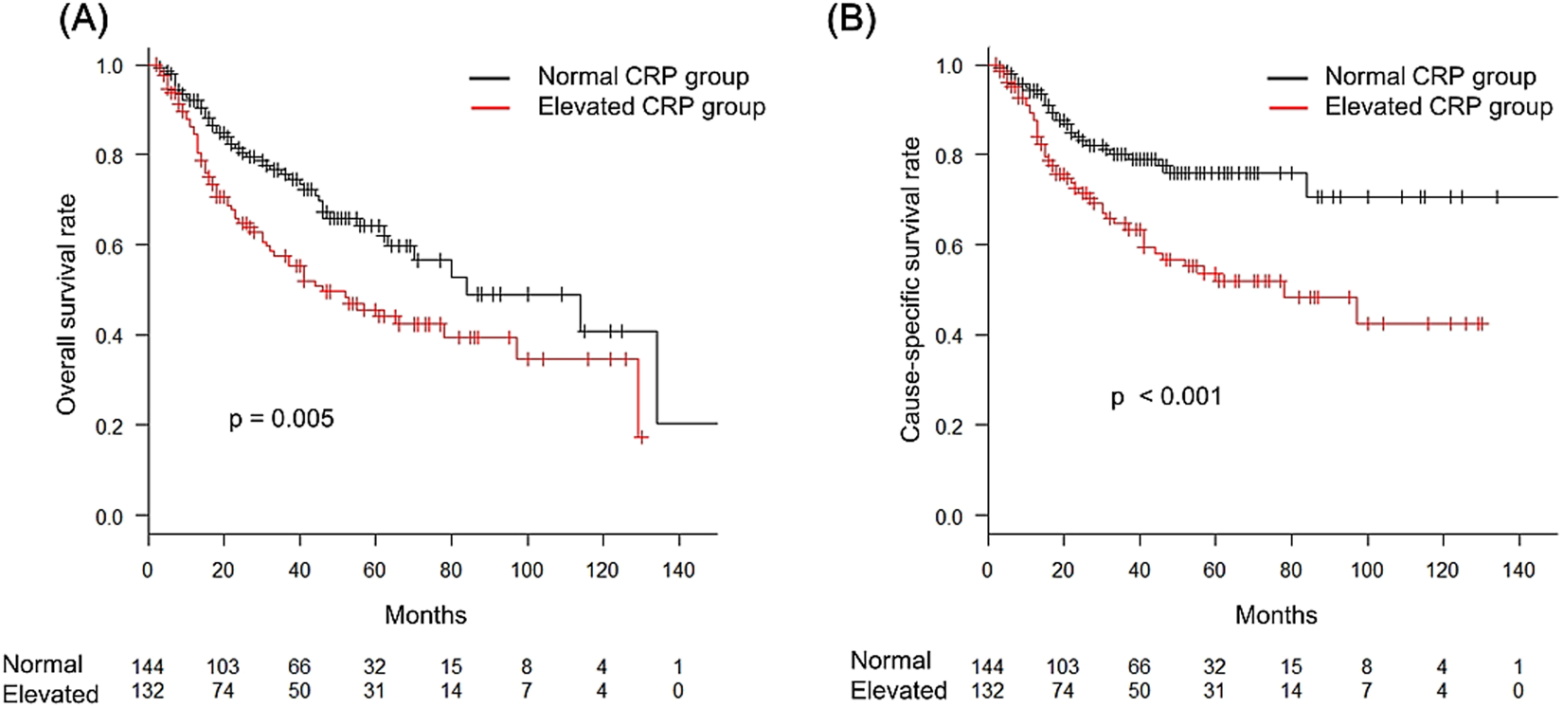
Kaplan-Meier curves for overall survival (**A**) and cause-specific survival (**B**). A vertical bar indicates a censored case.

The median follow-up for censored cases was 41 months (range: 2–171). The 3-year OS and CSS for all enrolled patients were 67.0% (95% CI: 60.5–72.7%) and 72.8% (95% CI: 66.4–78.2%), respectively. No significant differences were detected between oropharyngeal and hypopharyngeal cancer in OS (p = 0.69) and CSS (p = 0.71), as shown in Table 2. The OS and CSS rates were significantly worse in the elevated CRP group than in the normal CRP group, according to a comparison of Kaplan-Meier curves analysed by a Log-rank test (p = 0.005 and p < 0.001) (Fig. 1). Elevated CRP at pre-treatment was significantly correlated with advanced TNM staging by Fisher’s exact test (Supplementary Table 2). Relationship of pre-treatment CRP value and the same T or N stage of the treatment modalities were summarized in Supplementary Table 3. In a univariate analysis, KPS, smoking history, current smoking status, clinical T stage and absence of induction chemotherapy were also poorer prognostic factors in OS. KPS, smoking history, current smoking status, clinical T stage and clinical N stage were also poorer prognostic factors in CSS. A multivariate analysis revealed that pre-treatment serum CRP levels remained independent predictors for both OS (HR: 1.588, 95% CI: 1.07–2.36, p = 0.022) and CSS (HR: 1.989, 95% CI: 1.26–3.20, p = 0.005), regardless of other factors (Table 3).

**Table 2:** Univariate analysis of OS and CSS.

Variables		N	OS		CSS	
			3 year rate [95% CI]	p value*	3 year rate [95% CI]	p value*
Site of primary lesion	oropharynx	141	0.667 [0.576–0.744]	0.685	0.723 [0.632–0.795]	0.708
	hypopharynx	135	0.672 [0.574–0.752]		0.734 [0.638–0.808]	
Age	≤65 years	148	0.697 [0.608–0.769]	0.066	0.759 [0.672–0.825]	0.098
	>65 years	128	0.638 [0.537–0.722]		0.690 [0.589–0.771]	
Gender	Female	23	0.807 [0.563–0.923]	0.306	0.848 [0.596–0.949]	0.379
	Male	253	0.657 [0.588–0.717]		0.717 [0.649–0.774]	
KPS	≥90%	205	0.699 [0.625–0.761]	< 0.001	0.752 [0.680–0.811]	0.002
	<90%	71	0.582 [0.439–0.701]		0.656 [0.508–0.770]	
Current smoker	No	157	0.732 [0.644–0.801]	0.005	0.787 [0.701–0.851]	0.007
	Yes	119	0.590 [0.490–0.677]		0.650 [0.548–0.735]	
Smoking history	< 10 packs year	68	0.769 [0.633–0.860]	0.012	0.829 [0.696–0.908]	0.011
	≥ 10 packs year	208	0.639 [0.563–0.706]		0.697 [0.621–0.760]	
T stage	123	233	0.671 [0.600–0.733]	0.031	0.736 [0.666–0.794]	0.014
	4	43	0.668 [0.496–0.792]		0.690 [0.517–0.812]	
N stage	0	86	0.774 [0.656–0.856]	0.054	0.869 [0.761–0.930]	0.005
	123	190	0.624 [0.544–0.694]		0.666 [0.585–0.735]	
Hemoglobin Concentration	≤11g/dL	31	0.612 [0.408–0.764)]	0.110	0.797 [0.572–0.912]	0.612
	>11g/dL	245	0.678 [0.608–0.737]		0.721 [0.653–0.778]	
CRP	≤ 0.3 mg/dl	144	0.756 [0.668–0.823]	0.005	0.801 [0.716–0.862]	< 0.001
	>0.3 mg/dl	132	0.575 [0.477–0.662]		0.646 [0.545–0.730]	
IMRT	No	223	0.676 [0.605–0.736]	0.334	0.731 [0.662–0.789]	0.445
	Yes	53	0.655 [0.468–0.789]		0.728 [0.543–0.847]	
Radiation fractionation	Once-daily	244	0.664 [0.594–0.725]	0.704	0.730 [0.662–0.787]	0.795
	Other regimen*	32	0.719 [0.515–0.849]		0.719 [0.515–0.849]	
Neck dissection	No	235	0.676 [0.606–0.736]	0.857	0.732 [0.663–0.788]	0.889
	Yes	41	0.630 [0.432–0.775]		0.698 [0.489–0.835]	
Induction chemotherapy	No	182	0.645 [0.562–0.717]	0.046	0.701 [0.618–0.769]	0.139
	Yes	94	0.713 [0.602–0.799]		0.776 [0.668–0.853]	
Concurrent chemotherapy	No	164	0.697 [0.611–0.767]	0.263	0.759 [0.675–0.824]	0.140
	Yes	112	0.632 [0.527–0.720]		0.683 [0.577–0.768]	

*Other regimens include twice-daily and concomitant boost radiotherapy.

**Table 3:** Multivariate Cox regression analysis of OS and CSS.

Variables		OS		CSS	
		HR [95% CI]	p value	HR [95% CI]	p value
Age	>65 vs ≤ 65 years old	1.760 [1.176–2.635]	0.006	2.033 [1.261–3.279]	0.004
KPS	< 90 vs ≥ 90	1.755 [1.139–2.705]	0.011	1.696 [1.026–2.804]	0.039
Current smoking	Yes vs No	1.447 [0.939–2.230]	0.094	1.460 [0.880–2.420]	0.143
Smoking history	≥ 10 vs < 10 packs year	1.471 [0.822–2.632]	0.194	1.779 [0.853–3.711]	0.125
T stage	123 vs 4	1.263 [0.758–2.104]	0.371	1.495 [0.850–2.629]	0.163
N stage	123 vs 0	1.519 [0.968–2.383]	0.069	2.095 [1.170–3.752]	0.013
CRP	> 0.3 vs ≤ 0.3 mg/dl	1.588 [1.069–2.359]	0.022	1.989 [1.235–3.203]	0.005
Induction chemotherapy	Yes vs No	0.715 [0.463–1.103]	0.129	—	—

### Recurrence

**Figure 2:**
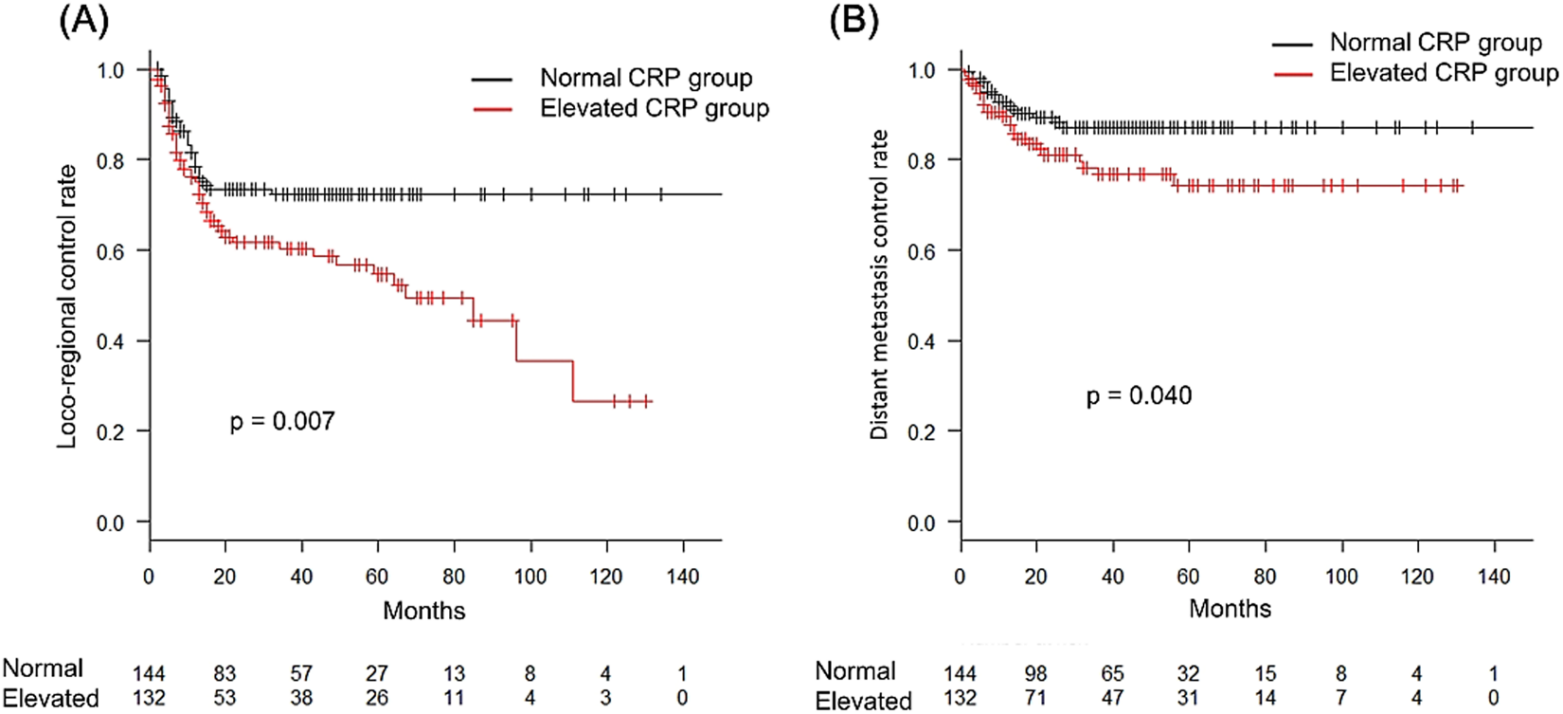
Kaplan-Meier curves for loco-regional control rate (**A**) and distant metastasis control rate (**B**). A vertical bar indicates a censored case.

A total of 112 patients (41%) developed recurrent disease. The first recurrence sites were located in loco-regional fields in 69 patients, distant metastatic fields in 18 patients, and both in 25 patients. We investigated factors predictive of loco-regional control (LRC) and distant metastasis control (DMC), respectively. The 3-year LRC and DMC for all enrolled patients were 66.8% (95% CI: 60.3–72.4%) and 82.4% (95% CI: 76.6–86.9%), respectively. The Kaplan–Meier analysis was used to calculate LRC and DMC rate curves. The log-rank test was used to detect significant differences between the elevated CRP group and the normal CRP group in LRC and DMC rate curves (p = 0.007 and p = 0.040, respectively) (Fig. 2, Supplementary Table 4). The LRC curve reached an approximate plateau after 5 years in the normal CRP group, but declined gradually in the elevated CRP group. In the multivariable analysis, elevated CRP levels remained as independent predictors for the LRC rate (HR: 1.52, 95% CI: 1.035–2.462, p = 0.034), but not for DMC (Supplementary Table 5). The significant determinants of DMC were N stage (HR: 3.35, 95% CI: 1.257–8.933, p = 0.016) and KPS (HR: 2.05, 95% CI: 1.076–3.900, p = 0.029).

## Discussion

This is the first study to show a significant correlation between elevated serum CRP levels and cancer prognosis in oro-hypopharynx cancer patients treated by radiotherapy. Moreover, our 3-year overall survival rate, progression-free survival rate and loco-regional control rate are comparable to those in previous reports^20^. Especially, it is curious that an elevated CRP serum level is a significant predictor of loco-regional recurrence. Nakamura *et al*. also reported the pre-operative CRP level as an independent predictor of local control^21^. Sun *et al*. reported that the tumor microenvironment plays an essential role in therapeutic resistance in cancer treatment^22^. In the present study, the pretreatment CRP elevation is significantly correlated with KPS and advanced clinical stage (Supplementary Table 2). These results are consistent with previous reports which reveals the positive relationship between CRP level and TNM tumor staging^7,11^.

The production of CRP is affected by inflammatory cytokines, such as interleukin-6 (IL-6), interleukin-1 (IL-1) and tumour necrosis factor (TNF)-alpha, which are secreted by monocytes or macrophages due to inflammation or cancer^23,24^. Especially, interleukin-6 is one of the multifunctional cytokines that control humoral immunity and are involved in inflammation, infection responses, and metabolic regulation^25^. Serum concentrations of IL-6 and CRP are positively correlated, and recent evidence suggests that IL-6 also affects cancer cell biology. It has been confirmed that an IL-6 signalling pathway stimulates cancer progression through the IL-6 receptor on the cancer cell surface in prostate cancer^25^. Shinriki *et al*. revealed that the IL-6 signalling system in human oral squamous cell carcinoma may be involved in the development of cancer by controlling angiogenesis and lymphangiogenesis^26^. In addition, the size and efficiency of tumor formation were dependent on IL-6 secretion in human ovarian cancer cells^27^. Other inflammatory cytokines, such as IL-1 and TNF-alpha, are also related to the immune-expression increases in cancer patients^28^. The prognostic relationship with serum CRP levels in cancer patients is probably a complicated and multifactorial process that is still not well understood, but it seems likely that inflammatory cytokines might mediate the relationship.

Recently, the relationship between serum CRP level and head and neck cancer was gradually uncovered. Zeng *et al*. reported that the degree of inflammation is a marker predicting the prognosis for 79 Patients with locally advanced nasopharyngeal carcinoma. They found that patients treated by chemo-radiotherapy had a poor prognosis if they had elevated serum CRP before treatment^29^. Multivariate analysis showed that CRP was an independent prognostic indicator of CSS, with a hazard ratio of 3.04 (95% confidence interval: 1.22 to 7.55; p = 0.017). Chen *et al*. analyzed pre-treatment combinations of serum squamous cell carcinoma antigen (SCC-Ag) and CRP levels in relation to prognosis for patients with pharyngo-laryngeal cancer^30^. They reported that elevated levels of SCC-Ag and CRP were associated with a high metabolic rate as well as proliferative activity measured by 18F-fluorodeoxyglucose positron emission tomography. In contrast, Astrid *et al*. reported no significant correlation between pre-treatment CRP levels and prognosis in patients with head and neck cancer treated by surgery^31^.

Elimination of inflammation might be an effective strategy in cancer treatment. The mechanisms underlying the effects of anti-inflammatory drugs are gradually being elucidated. Wang *et al*. reported that long-term oral administration of non-steroidal anti-inflammatory drugs such as aspirin significantly reduced the risk of carcinogenesis leading to colorectal cancer^32^. Administration of non-steroidal anti-inflammatory drugs could be beneficial in enhancing chemotherapy effects in conventional cancer treatment^33^. Although it is probably premature to begin using these drugs at the present time to enhance cancer treatment, anti-inflammatory drugs might bring new insight into cancer therapy. Many studies are still necessary in order to elucidate which lesion or cell type is suitable for cancer prevention or treatment with anti-inflammatory agents.

There were several limitations of our study to mention. First, this was a retrospective analysis with a limited number of cases and possible selection bias because of the retrospective nature of the study. Second, patients were evaluated during considerable long period, which was correlated with various treatment strategy regarding radiation technique and chemotherapy regimens. In addition, the ambiguities about criteria of the different treatment strategies could not be resolved. Despite a large variety of treatments were applied, relatively low patient number was included. Moreover, sufficient relevant prognostic factors were not considered in the statistical analysis. Third, the each clinical endpoint (OS, CSS, LRC and DMC) was not fully independent of each other, type I error inflation might have existed. Fourth, limited information was available regarding pretreatment patients’ characteristic. We need further consideration of relevant factors that affect the pre-treatment CRP value.

In conclusion, the pre-treatment CRP level was an independent predictor of prognosis in patients with oro-hypopharyngeal squamous cell carcinoma undergoing definitive radiotherapy. An elevated CRP level might be useful to identify candidates for more active surveillance and potential adjuvant therapy. However, since the sample size in this study was limited, a confirmatory prospective cohort study with a larger number of patients and a long follow-up period should be carried out. In addition, treatment responsiveness should be stratified in the patients’ background information.

## Electronic supplementary material

SupplementaryData1
