# Supplementary material for: The impact of elevated C-reactive protein level on the prognosis for oro-hypopharynx cancer patients treated with radiotherapy
Source: Sci Rep. 2017 Dec 19;7:17805. doi: 10.1038/s41598-017-18233-w (PMC5736698; doi:10.1038/s41598-017-18233-w)
Supplement: Supplementary file 1 — SupplementaryData1 [file 41598_2017_18233_MOESM1_ESM.doc]

**The impact of elevated C-reactive protein level on the prognosis for oro-hypopharynx cancer patients treated with radiotherapy**

--------Supplementary Data------------

Atsuto Katano^1^, Wataru Takahashi^1^, Hideomi Yamashita^1*^, Kentaro Yamamoto^2^, Mizuo Ando^3^, Masafumi Yoshida^3^, Yuki Saito^3^, Osamu Abe^1^ and Keiichi Nakagawa^1^

1. Department of Radiology, The University of Tokyo, 7-3-1 Hongo, Bunkyo-ku, Tokyo, 113-8655, Japan

2. Department of Radiology, Japan Self Defense Force Central Hospital, 1-2-24 Ikejiri, Setagaya-ku, Tokyo, 154-8532, Japan

3. Department of Otolaryngology - Head and Neck Surgery, The University of Tokyo, 7-3-1 Hongo, Bunkyo-ku, Tokyo, 113-8655, Japan

* Corresponding author: Hideomi Yamashita

Department of Pathology, the University of Tokyo Hospital, Tokyo, Japan

Address: 7-3-1 Hongo, Bunkyo-ku, Tokyo, 113-8655, Japan

Telephone: +81-3-5800-8667

E-mail: yamachan07291973@yahoo.co.jp

Supplementary Table 1. Patient distribution according to clinical stage for each cancer

| T stage |  |  |
| --- | --- | --- |
|  | N (percent) | |
|  | oropharynx | hypopharynx |
| T1 | 17 (12) | 24 (18) |
| T2 | 56 (40) | 57 (42) |
| T3 | 41 (29) | 38 (28) |
| T4 | 27 (19) | 16 (12) |
| Total | 141 (100) | 135 (100) |
|  |  |  |
| N stage |  |  |
|  | N (percent) | |
|  | oropharynx | hypopharynx |
| N0 | 39 (28) | 47 (35) |
| N1 | 20 (14) | 21 (16) |
| N2a | 12 (9) | 8 (6) |
| N2b | 35 (25) | 44 (33) |
| N2c | 19 (13) | 8 (6) |
| N3 | 16 (11) | 7 (5) |
| Total | 141 (100) | 135 (100) |

Supplementary Table 2. Relationship of patient characteristic and pre-treatment CRP level

|  | | N (percent) or Median (range) | |  |
| --- | --- | --- | --- | --- |
|  |  | normal CRP  [≤ 0.3 mg/dl)] | elevated CRP  [> 0.3 mg/dl)] | p value |
| Site of primary lesion | |  |  |  |
|  | Hypopharynx | 70 (51) | 65 (51) | 1 |
|  | Oropharynx | 74 (49) | 67 (49) |  |
| Age | | 64 (40-88) | 65 (21-93) | 0.907 |
| Gender | |  |  |  |
|  | Male | 130 (90) | 123 (93) | 0.514 |
|  | Female | 14 (10) | 9 (7) |  |
| Karnofsky Performance Scale | | 90 (70-100) | 90 (50-100) | 0.015 |
| Current smoker | |  |  |  |
|  | Yes | 56 (39) | 63 (48) | 0.146 |
|  | No | 88 (61) | 69 (52) |  |
| Smoking history (packs year) | | 30 (0-150) | 36 (0-141) | 0.098 |
| Clinical T stage | |  |  |  |
|  | 4 | 15 (10) | 28 (21) | 0.003 |
|  | 3 | 34 (24) | 45 (34) |  |
|  | 2 | 68 (47) | 45 (34) |  |
|  | 1 | 27 (19) | 14 (11) |  |
| Clinical N stage | |  |  |  |
|  | 3 | 6 (4) | 17 (13) | 0.032 |
|  | 2 | 64 (44) | 62 (47) |  |
|  | 1 | 22 (15) | 19 (14) |  |
|  | 0 | 52 (36) | 34 (26) |  |

Supplementary Table 3. Relationship of pre-treatment CRP value and the same T or N stage of the induction chemotherapy and/or concurrent chemotherapy.

| T stage |  | IC+RT | CCRT | IC+CCRT | RT alone | p value |
| --- | --- | --- | --- | --- | --- | --- |
| T1 |  |  |  |  |  |  |
|  | normal CRP (≤ 0.3 mg/dl) | 4 | 2 | 0 | 21 | 0.210 |
|  | elevated CRP (> 0.3 mg/dl) | 1 | 4 | 0 | 9 |  |
| T2 |  |  |  |  |  |  |
|  | normal CRP (≤ 0.3 mg/dl) | 30 | 9 | 2 | 27 | 0.005 |
|  | elevated CRP (> 0.3 mg/dl) | 12 | 16 | 5 | 12 |  |
| T3 |  |  |  |  |  |  |
|  | normal CRP (≤ 0.3 mg/dl) | 8 | 16 | 5 | 5 | 0.604 |
|  | elevated CRP (> 0.3 mg/dl) | 15 | 20 | 3 | 7 |  |
| T4 |  |  |  |  |  |  |
|  | normal CRP (≤ 0.3 mg/dl) | 6 | 5 | 1 | 3 | 0.002 |
|  | elevated CRP (> 0.3 mg/dl) | 1 | 23 | 1 | 3 |  |
| Total |  | 77 | 95 | 17 | 87 |  |
|  |  |  |  |  |  |  |
|  |  |  |  |  |  |  |
| N stage |  | IC+RT | CCRT | IC+CCRT | RT alone | p value |
| N0 |  |  |  |  |  |  |
|  | normal CRP (≤ 0.3 mg/dl) | 12 | 5 | 1 | 34 | 0.012 |
|  | elevated CRP (> 0.3 mg/dl) | 8 | 12 | 1 | 13 |  |
| N1 |  |  |  |  |  |  |
|  | normal CRP (≤ 0.3 mg/dl) | 11 | 5 | 2 | 4 | 0.963 |
|  | elevated CRP (> 0.3 mg/dl) | 8 | 6 | 1 | 4 |  |
| N2 |  |  |  |  |  |  |
|  | normal CRP (≤ 0.3 mg/dl) | 24 | 20 | 3 | 17 | 0.014 |
|  | elevated CRP (> 0.3 mg/dl) | 10 | 34 | 5 | 13 |  |
| N3 |  |  |  |  |  |  |
|  | normal CRP (≤ 0.3 mg/dl) | 1 | 2 | 2 | 1 | 0.360 |
|  | elevated CRP (> 0.3 mg/dl) | 3 | 11 | 2 | 1 |  |
| Total |  | 77 | 95 | 17 | 87 |  |

IC: induction chemotherapy, RT: radiotherapy, CCRT: concurrent chemotherapy

Supplementary Table 4. Univariate analysis of LRC and DMC.

| Variables |  |  | 3 year LRC rate | |  | 3 year DMC rate | |
| --- | --- | --- | --- | --- | --- | --- | --- |
|  |  | N | HR [95% CI] | p value* |  | HR [95% CI] | p value* |
| Site of primary lesion | oropharynx | 141 | 0.715 [0.626-0.786] | 0.074 |  | 0.849 [0.773-0.902] | 0.773 |
|  | hypopharynx | 135 | 0.616 [0.518-0.700] |  |  | 0.794 [0.697-0.863] |  |
| Age | ≤65 years | 148 | 0.666 [0.577-0.740] | 0.927 |  | 0.834 [0.755-0.889] | 0.887 |
|  | >65 years | 128 | 0.668 [0.567-0.750] |  |  | 0.810 [0.714-0.877] |  |
| Gender | Female | 23 | 0.790 [0.529-0.917] | 0.231 |  | 0.906 [0.673-0.976] | 0.346 |
|  | Male | 253 | 0.656 [0.588-0.716] |  |  | 0.816 [0.753-0.863] |  |
| KPS | ≥90% | 205 | 0.705 [0.633-0.765] | 0.063 |  | 0.866 [0.806-0.908] | 0.005 |
|  | <90% | 71 | 0.509 [0.348-0.648] |  |  | 0.672 [0.503-0.795] |  |
| Current smoking | No | 157 | 0.733 [0.648-0.801] | 0.002 |  | 0.843 [0.763-0.898] | 0.110 |
|  | Yes | 119 | 0.577 [0.474-0.667] |  |  | 0.801 [0.709-0.867] |  |
| Smoking history | < 10 packs year | 68 | 0.866 [0.748-0.931] | <0.001 |  | 0.891 [0.769-0.950] | 0.119 |
|  | ≥ 10 packs year | 208 | 0.604 [0.527-0.672] |  |  | 0.802 [0.732-0.856] |  |
| T stage | 12 | 233 | 0.686 [0.616-0.746] | 0.051 |  | 0.829 [0.766-0.876] | 0.644 |
|  | 34 | 43 | 0.563 [0.383-0.710] |  |  | 0.794 [0.608-0.899] |  |
| N stage | 0 | 86 | 0.794 [0.685-0.869] | 0.033 |  | 0.942 [0.850-0.978] | 0.003 |
|  | 123 | 190 | 0.608 [0.526-0.680] |  |  | 0.769 [0.691-0.829] |  |
| Hemoglobin Concentration | ≤11g/dL | 31 | 0.628 [0.401-0.789] | 0.691 |  | 0.875 [0.654-0.959] | 0.429 |
|  | >11g/dL | 245 | 0.673 [0.605-0.732] |  |  | 0.817 [0.755-0.865] |  |
| CRP | ≤ 0.3 mg/dl | 144 | 0.722 [0.635-0.792] | 0.007 |  | 0.872 [0.798-0.920] | 0.040 |
|  | >0.3 mg/dl | 132 | 0.602 [0.501-0.689] |  |  | 0.766 [0.667-0.840] |  |
| IMRT | No | 223 | 0.683 [0.613-0.743] | 0.366 |  | 0.846 [0.785-0.890] | 0.106 |
|  | Yes | 53 | 0.577 [0.386-0.727] |  |  | 0.673 [0.455-0.819] |  |
| Radiation fractionation | Once-daily | 244 | 0.656 [0.586-0.717] | 0.460 |  | 0.828 [0.766-0.875] | 0.617 |
|  | Other regimen* | 32 | 0.750 [0.543-0.873] |  |  | 0.788 [0.586-0.900] |  |
| Neck dissection | No | 235 | 0.652 [0.582-0.714] | 0.186 |  | 0.840 [0.779-0.886] | 0.124 |
|  | Yes | 41 | 0.747 [0.534-0.873] |  |  | 0.711 [0.491-0.849] |  |
| Induction chemotherapy | No | 182 | 0.654 [0.572-0.725] | 0.218 |  | 0.797 [0.719-0.855] | 0.091 |
|  | Yes | 94 | 0.692 [0.580-0.780] |  |  | 0.874 [0.777-0.931] |  |
| Concurrent chemotherapy | No | 164 | 0.690 [0.605-0.761] | 0.196 |  | 0.866 [0.791-0.916] | 0.010 |
|  | Yes | 112 | 0.633 [0.528-0.721] |  |  | 0.762 [0.661-0.836] |  |

* Other regimens include twice-daily and concomitant boost

Supplementary Table 5. Multivariate Cox regression analysis of LRC and DMC

| Variables |  | LRC | |  | DMC | |
| --- | --- | --- | --- | --- | --- | --- |
|  |  | HR [95% CI] | p value |  | HR [95% CI] | p value |
| primary site | oropharynx vs hypopharynx | 1.414 [0.915-2.186] | 0.119 |  | - | - |
| KPS | < 90 vs ≥ 90 | 1.238 [0.756-2.028] | 0.397 |  | 2.048 [1.076-3.900] | 0.029 |
| Current smoking | Yes vs No | 1.227 [0.776-1.938] | 0.382 |  | - | - |
| Smoking history | ≥ 10 vs < 10 packs year | 2.746 [1.309-5.761] | 0.008 |  | - | - |
| T stage | 123 vs 4 | 1.261 [0.721-2.206] | 0.415 |  | - | - |
| N stage | 123 vs 0 | 1.523 [0.920-2.522] | 0.102 |  | 3.351 [1.257-8.933] | 0.016 |
| CRP | > 0.3 vs ≤ 0.3 mg/dl | 1.596 [1.035-2.462] | 0.034 |  | 1.387 [0.718-2.682] | 0.330 |
| Induction chemotherapy | Yes vs No | - | - |  | 0.630 [0.279-1.421] | 0.265 |
| Concurrent chemotherapy | Yes vs No | - | - |  | 1.328 [0.637-2.768] | 0.449 |
